# Evaluation of Clinical and Immune Responses in Recovered Children with Mild COVID-19

**DOI:** 10.3390/v14010085

**Published:** 2022-01-04

**Authors:** Xiaodong Tian, Zhihua Bai, Ying Cao, Haizhou Liu, Di Liu, Wenjun Liu, Jing Li

**Affiliations:** 1 CAS Key Laboratory of Pathogenic Microbiology and Immunology, Institute of Microbiology, Chinese Academy of Sciences, Beijing 100101, China; tienhsiaotung@foxmail.com (X.T.); baizhihua19@mails.ucas.ac.cn (Z.B.); 2 School of Life Sciences, University of Science and Technology of China, Hefei 230026, China; 3 Savaid Medical School, University of the Chinese Academy of Sciences, Beijing 100049, China; 4 Computational Virology Group, Center for Bacteria and Virus Resources and Application, Wuhan Institute of Virology, Chinese Academy of Sciences, Wuhan 430071, China; caoyingor@163.com (Y.C.); liuhz@wh.iov.cn (H.L.)

**Keywords:** SARS-CoV-2, recovered children, clinical, immune responses, mild COVID-19

## Abstract

The coronavirus disease 2019 (COVID-19) has spread globally and variants continue to emerge, with children are accounting for a growing share of COVID-19 cases. However, the establishment of immune memory and the long-term health consequences in asymptomatic or mildly symptomatic children after severe acute respiratory syndrome coronavirus 2 infection are not fully understood. We collected clinical data and whole blood samples from discharged children for 6–8 months after symptom onset among 0-to-14-year-old children. Representative inflammation signs returned to normal in all age ranges. The infants and young children (0–4 years old) had lung lesions that persisted for 6–8 months and were less responsive for antigen-specific IgG secretion. In the 5-to-14-year-old group, lung imaging abnormalities gradually recovered, and the IgG-specific antibody response was strongest. In addition, we found a robust IgM^+^ memory B cell response in all age. Memory T cells specific for the spike or nucleocapsid protein were generated, with no significant difference in IFN-γ response among all ages. Our study highlights that although lung lesions caused by COVID-19 can last for at least 6–8 months in infants and young children, most children have detectable residual neutralizing antibodies and specific cellular immune responses at this stage.

## 1. Introduction

Coronavirus disease 2019 (COVID-19) caused by severe acute respiratory syndrome coronavirus 2 (SARS-CoV-2) is wreaking havoc worldwide [1,2,3]. Detecting the effectiveness and durability of the immunologic response to SARS-CoV-2 in convalescent persons will allow better assessment of the risk of reinfection and formulation of vaccination strategies. SARS-CoV-2 elicits the production of broadly directed and functionally replete memory T cells with different functions [4,5]. Specific antibodies capable of neutralizing the virus persist for at least 9 months in most recovered individuals, and antigen-specific IgG^+^ memory B cells increase in number during recovery [6,7]. Importantly, memory lymphocytes from COVID-19 patients display functional responsiveness that may contribute to antiviral resistance upon reinfection [8]. The available data have demonstrated that both humoral and cellular immunity are involved in COVID-19 recovery and may protect against recurrent episodes of severe COVID-19 [9,10]. Nevertheless, it is still worth emphasizing that rather than a definitive picture, the understanding of adaptive immunity to SARS-CoV-2 is still evolving.

From the quotable data, children constitute a growing share of COVID-19 cases. More than 3.87 million children (0–15 years old) have tested positive since the outbreak began, accounting for approximately 11.6% of all cases in the United States (https://covid.cdc.gov/covid-data-tracker/, accessed on 29 November 2021). Epidemiological investigations and clinical monitoring indicate that the majority of infected children tend to develop mild or asymptomatic symptoms [11,12,13,14]. However, there is still little information about the magnitude or stability of the immune response in this large population and whether the development of immune memory varies depending on the age of the individual or the severity of the disease.

In response to this need, we recruited 31 convalescent children who had asymptomatic or mildly symptomatic COVID-19 between 27 January and 11 March 2020 and described the recovery situation after discharge at the 6–8-month revisit. We collected serum and PBMCs, focused on evaluating the dynamics of specific antibodies and measured the antigen-specific memory B cell and T cell responses for up to 6–8 months after acute infection. We also analyzed the correlation between the humoral and cellular immune responses and individual age at infection. Knowledge of the durability of the initial immune response and the protective capacity of immune memory will provide references for the protection for children and provide a basis for future vaccine development for children.

## 2. Methods and Materials

### 2.1. Ethics Statement

The analytical samples and protocols used in this study were approved by the Ethics Committee of Wuhan Children’s Hospital and Wuhan Maternal and Child Health Hospital (Approval Code: WHCH2020003, Approval Date: 4 February 2020). A written statement that the formal consent of the parent/guardian has been obtained and that the parent/guardian is informed that the study is anonymous. All experiments involving SARS-CoV-2 strains were conducted in a biosafety level 2 (BSL2) laboratory, were approved by the Institute of Microbiology, Chinese Academy of Sciences (IMCAS), and complied with all relevant ethical regulations regarding human research.

The HEK-293T cells line was provided by *ATCC* CRL-11268. The 293T-ACE2 cell line, the pLenti-GFP lentiviral reporter, plasmids psPAX2, and codon-optimized cDNA encoding SARS-CoV-2 S glycoprotein (QHU36824.1) were obtained from Dr. Zhao Zhendong, Institute of Pathogenic Biology, Chinese Academy of Medical Sciences [15].

### 2.2. Study Design

Serum samples (n = 31) and PBMCs (n = 21) of 31 recovered children (RC) were collected 6–8 months after initial diagnosis. Serum samples (n = 22) and PBMCs (n = 17) were isolated from 22 age-matched healthy controls (HC). Anti-Spike protein antibody and anti-Nucleocapsid protein antibody (IgG and IgM) levels were measured by ELISA to assess serum antibody levels during recovery, and T/B cells, NK cells and monocytes were further divided by flow cytometry to determine the memory subtypes of T cells and B cells and to interpret the effects of SARS-CoV-2 infection on the immune system. In addition, to evaluate the functional status of T cell and B cell populations during recovery, PBMCs were stimulated in vitro to detect the secretion levels of cytokines (IFN-γ) and antibodies (IgG, IgM, and IgA). A comprehensive analysis of the above results revealed the characteristics of adaptive immune memory in children who recovered from COVID-19 and its correspondence with clinical signs.

### 2.3. Collection of Clinical Samples from Children

The samples were collected from recovered children in Wuhan Children’s Hospital, with a mean ± standard deviation (STDEV) and age range of 5.48 ± 4.48 and 0.1–14, respectively. In the healthy control group, the mean ± STDEV and age ranges were 5.09 ± 3.54 and 0.58–12, respectively. The patient infections were confirmed by one or more RT-PCR tests. The clinical diagnosis was based on the Diagnosis, Treatment and Prevention of 2019 Novel Coronavirus Infection in Children: Experts’ Consensus Statement (Third Edition). Asymptomatic infected persons were defined as those who had no self-perceived or clinically identifiable symptoms or signs of upper respiratory tract infection, namely, pharyngeal congestion, sore throat and fever, or abnormalities on imaging examination. Mild infection was defined as a child with pneumonia with radiological respiratory symptoms.

Whole blood was diluted with an equal amount of PBS and transferred to a SepMate tube (STEMCELL Technologies). Serum, platelets, granulocytes, and red blood cells were isolated from PBMCs using Lymphoprep^TM^ (STEMCELL Technologies) and centrifuged at 1000× *g* at 20 °C for 10 min. The PBMCs were transferred and washed with PBS and centrifuged at 350× *g* for 5 min at room temperature. PBMCs were resuspended and eventually stored in liquid nitrogen until use. The serum was precipitated with coagulant, inactivated, and stored at −80 °C before use.

### 2.4. Cell Culture

The frozen PBMCs were thawed at 37 °C and prepared in Roswell Park Memorial Institute (RPMI) 1640 medium containing nuclease (final concentration ≥ 50 U/mL) for 30 min. After centrifugation at 1000× *g* for 5 min at room temperature, the cells were washed twice with RPMI 1640 medium. Finally, the nuclei of PBMCs were stained with Acridine Orange/Propyl Iodide (AO/PI) dye, and the ratio of dead cells to alive cells was calculated in the fluorescence field. In the fluorescence field, the nuclei of dead cells were red and those of living cells were green.

The HEK-293T cell line and 293T-ACE2 cell line were cultured in Dulbecco’s modified Eagle’s medium (DMEM) containing 10% fetal bovine serum (Gibco) and 100 μg/mL penicillin-streptomycin (Gibco) in 5% CO_2_ at 37 °C.

### 2.5. ELISA

Binding plates (Costar) were coated with 100 ng/well Spike protein or Nucleocapsid protein (SinoBiological) at 4 °C overnight. The plates were blocked with 4% bovine serum albumin (BSA) for 90 min. After blocking, 50 μL of serum dilution was added to each well and incubated at 37 °C for 1 h. The plates were washed with PBS containing 0.05% Tween-20. Enzyme-labeled goat anti-human IgG-Fc or enzyme-labeled goat anti-human IgM-Fc was added as the secondary antibody. After incubation for 45 min, the plates were washed, 100 μL of 3,3′,5,5′-tetramethylbenzidine (TMB) substrate was added, and then 100 μL of 2 mol/L H_2_SO_4_ was added to stop the reaction. The microplates were read using a microplate reader (Multiskan FC, Thermo Scientific) at 450 nm. The mean HC area under the curve (AUC) value + 3SD was used as the cutoff value to determine positive serum samples [6].

### 2.6. Pseudovirus Neutralization Assays (PNT_50_)

Serum neutralizing antibodies (NAbs) in children with mild COVID-19 were detected by PNT_50_ assays, as described previously [15]. 293T cells were cotransfected with psPAX2, plenti-GFP and Spike protein to obtain pseudoviruses. 293T-ACE2 cells were seeded into 96-well plates at a density of 5 × 10^4^ cells/well 24 h before infection. The diluent was mixed with a certain amount of pseudovirus, neutralized for 1 h, added to a 96-well plate, and incubated at 37 °C for 48 h. The fate of the cells was monitored with high sensitivity using luciferase-based gene reporters (Beyotime). Finally, the semineutralization concentration was calculated by fitting the four-parameter curves according to the fluorescence values of different dilutions.

### 2.7. Flow Cytometry and Antibodies

PBMCs were used for cell composition analysis. Cells were stained with appropriate antibody–fluorophore conjugates in cold fluorescence-activated cell sorting (FACS) buffer at 4 °C for 30 min after incubation with Fc Block (BD Biosciences) and analyzed on an ARIA II (BD Biosciences). Dead cells were excluded via viability dye (eBioscience) staining for 30 min. Data were analyzed with FlowJo software (BD Biosciences). Those for lymphocyte populations (FSC-H and SSC-H), (SSC-A and SSC-H), and the selection of living leukocytes (Fixable Viability Dye-780^−^ and CD45- PE-eflour610^+^). After the exclusion of monocytes (CD14-eflour450^+^) and NK cells (Cd56-PE-Cyanine7^+^), T cells, memory T cells (CD3-eflour560^+^CD4-FITC^+^/CD8-PE-Cyanine5.5^+^, CCR7-PE^+^, and CD45RA-APC^+^), B cells, and memory B cells (CD19-PerCP-Cyanine5.5^+^ and CD27-Super Bright 780^+^) were analyzed. All above antibodies were obtained from eBioscience

### 2.8. ELISPOT

According to the manufacturer’s instructions, 50 μL of a 70% ethanol/water solution was added to the precoated IFN-γ board and incubated for 2 min, the polyvinylidene fluoride membrane was activated at the bottom of the board, and the membrane was rinsed 5 times with sterile water. A total of 200,000 white blood cells were mixed with S or N protein (10 μg/mL). An anti-CD3 antibody was added as positive control. The boards were incubated for 48 h in 5% CO_2_ at 37 °C and then removed. After washing, an HRP-conjugated antibody and TMB substrate were added for color rendering. When obvious spots appeared, the entire plate was rinsed with deionized water to stop the reaction. During the ELISPOT assays, 0–1 points were observed for each well in the control group.

### 2.9. FluoroSpot Assays

According to the manufacturer’s instructions, S or N protein was precoated on an IPFL plate as an antigen. The number of thawed PBMCs was adjusted and counted, and the cells were prestimulated with R848 and IL-2 in 48-well plates for 72 h. Finally, the cells were transferred to plates coated with S or N protein and incubated again for 24 h. The plates were removed and washed, and a 1:500-diluted antibody mixture was added and incubated. The fluorescence enhancer was incubated at 50 μL/well for 15 min at room temperature, and then the plates were washed and dried to obtain clearer fluorescence spots. Fluorescent spots were recorded with a Fluorescent Spot Reader (AID iSpot Spectrum, AID GmbH).

### 2.10. Statistical Analysis

The t-distributed stochastic neighbor embedding (tSNE) dimension reduction analysis was used to visualize the molecular expression patterns on the labeled cell surface. All of the data are presented as the mean ± standard error of the mean. Spearman rank-correlation tests were used for correlation analysis (ρ), and the Mann–Whitney U-test was used to determined *p* value to compare position measurements of the healthy children and recovered groups with questionable normality assumptions.

## 3. Results

### 3.1. Clinical Characteristics of COVID-19 Convalescent Subjects

To understand the long-term health consequences of COVID-19 in children, we collected and compared 31 patients’ clinical information and data during the acute phase of infection and approximately 6–8 months after symptom onset. Detailed information is shown in Supplementary Table S1. Among all individuals, 18 patients experienced mild pneumonia (58%), 11 patients presented with acute upper respiratory tract infection (AURI) (36%), and 2 confirmed persons were asymptomatic (6%). As shown in Table 1, in contrast with those in other age groups, most patients in the 0–4-year-old group developed mild pneumonia (63%). Furthermore, mild elevations in the levels of inflammatory markers such as C-reactive protein (CRP), procalcitonin (PCT), and D-dimer, a biomarker associated with COVID-19 disease severity, returned to the normal range at follow-up, as well as those of other serological markers of the patients (Supplementary Table S2).

We also observed lung tissue repair and respiratory function recovery. All patients completed both pulmonary function tests and chest CT imaging during the follow-up. The lung imaging abnormalities gradually improved over 6–8 months. In most patients, the proportions of ground-glass opacities decreased, and lung lesions were absorbed. However, patchy shadows were still seen in several cases. Additionally, pulmonary function tests showed no pulmonary dysfunction or mildly restrictive lesions (33%) in the 5–9-year-old or 10–14-year-old age group, respectively. In the 0-to-4-year-old age group, 13% of patients presented mild-to-moderate obstructive disease, and 25% of patients presented moderate obstructive disease (Supplementary Table S3). Overall, sequential follow-up evaluations at 6–8 months after COVID-19 onset demonstrated a vast improvement in symptoms and chest CT abnormalities over time, and the majority of study participants had functional recovery from COVID-19. However, we also found that some children, especially infants and young children, had lung lesions that persisted for 6–8 months.

### 3.2. SARS-CoV-2-Specific Antibodies Could Be Detected but Their Levels Waned in Recovered Children

**Figure 1:**
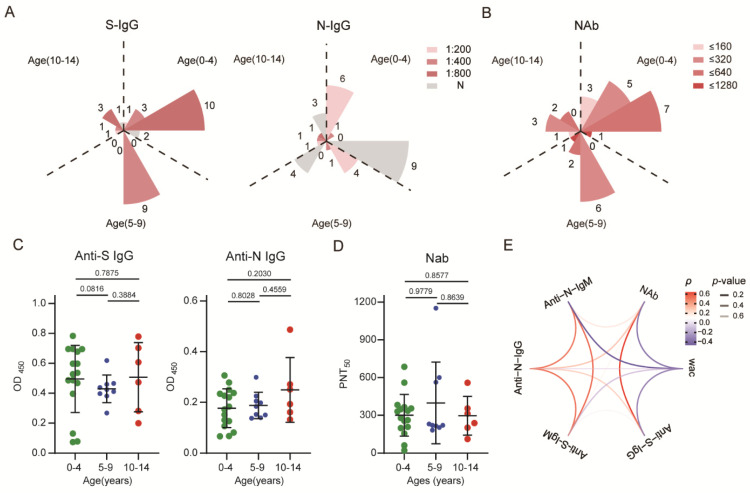
SARS-CoV-2-specific antibody response in children recovered for 6–8 months: (**A**) The absolute positive numbers of individuals with anti-S IgG and anti-N IgG antibody titers of un-detected (N), 1:200 (low), 1:400 (moderate), and 1:800 (high). (**B**) Neutralizing antibody titers were determined according to different dilutions. (**C**,**D**) Differences in antibody levels between different age groups. (**E**) Correlations between different antibodies and the correlation between antibodies over time. Red indicates a positive correlation, blue indicates a negative correlation, and transparency represents a change in the *p* value; the greater the *p* value is, the greater the line transparency. Each dot represents one donor. Data were analyzed using unpaired, two-tailed *t*-tests (ns, *p* > 0.05). Error bars represent the mean and SD. RC, recovered children; HC, healthy control; WAC, weeks after confirmation; S, spike; N, nucleocapsid.

To evaluate the persistence of the antibody response in recovered children with mild COVID-19, the levels of SARS-CoV-2-specific spike or nucleocapsid antibodies in serum were detected. The results showed that recovered children who had all of these antibodies accounted for 3% of the total samples; those who were S-IgG^+^ and N-IgG^+^ accounted for 38.7%; those with only S-IgG^+^ accounted for 48.4%; and the percentage of those who were S-IgG-, S-IgM-, N-IgG^+^, and N-IgM^+^ was 3%. Specifically, the positive rates for specific anti-S IgG and anti-N IgG were 81.3% and 43.8% in 0-to-4-year-old children, 100% and 44.4% in 5-to-9-year-old children, and 83.3% and 50% in 10-to-14-year-old children, respectively (Supplementary Figure S1A,B). Furthermore, to compare the levels of residual antibodies in the antibody-positive population, the highest serum dilution <200 was defined as low, between 200 and 400 as moderate, and between 400 and 800 as high. Anti-S protein IgG levels were primarily maintained at a high level (71.4%) and moderate level (100%) in the 0–4-year-old and 5–9-year-old age groups, respectively, while those of anti-N protein IgG were maintained mainly at low levels in both groups (85.7% and 80.0%), respectively. In the 10-to-14-year-old age group, anti-S protein IgG was mainly maintained at a high level (60.0%), and anti-N protein IgG was evenly distributed among the three titers (Figure 1A). The data regarding subsequent detection of serum NAb levels are shown in Supplementary Figure S1E. The examined children with PNT50 in the range of 160–640 accounted for the majority of all ages, with rates of 75% (0–4), 89% (5–9), and 83% (10–14), respectively (Figure 1B). Additionally, 97% of the recovered children had a negative conversion of anti-S IgM, and 94% had a negative conversion of anti-N IgM. Only extremely low levels of anti-S and anti-N IgM were observed in a few individuals (Supplementary Figure S1C,D).

There was no significant difference in the activities of serum-specific antibodies and NAbs among groups of different ages (Figure 1C,D). In addition, NAb titers showed a correlation with anti-S IgG levels (*p* = 0.6743, *p* < 0.0001) but no significant correlation with other antibodies. Similar to the dynamics of anti-S and anti-N specific antibody levels, the neutralizing activity of plasma antibodies showed a negative correlation with weeks since diagnosis confirmation, decreasing gradually over time (Figure 1E). Collectively, the results indicated that children with mild COVID-19 who fully recovered produced a specific antibody response to SARS-CoV-2. Despite a rapid decline in IgM antibody levels, IgG antibodies and plasma neutralizing activity could be detected at least 6–8 months after diagnosis. Additionally, the level of S protein-specific antibodies remained higher than that of N protein-specific antibodies (Figure 1A).

### 3.3. The Immune Response Returns to Homeostasis in Recovered Children at 8 Months

**Figure 2:**
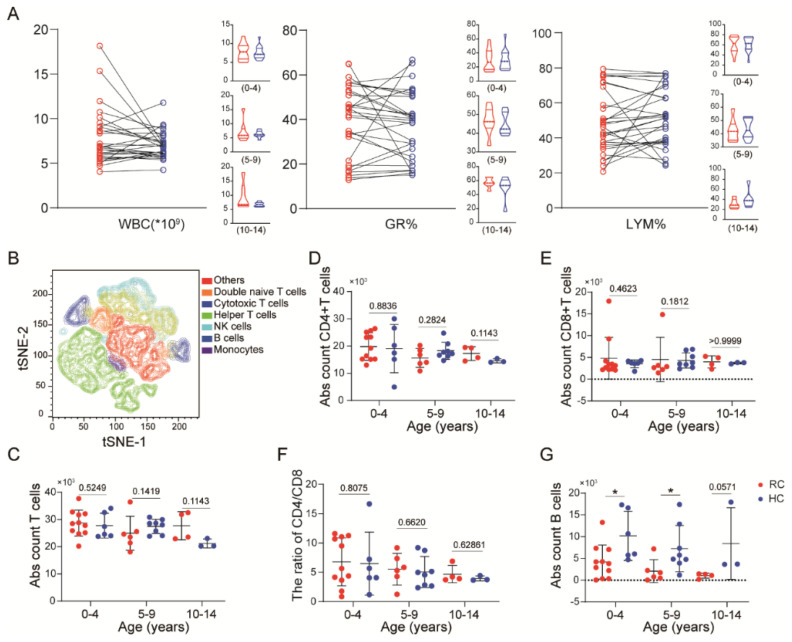
The immune response returns to homeostasis in children with mild COVID-19 at 8 months: (**A**) Changes in white blood cells (WBC), neutrophils and lymphocytes percentages after acute infection in children. (**B**) The tSNE plots shows the clustering of PBMCs. (**C**–**E**) The absolute counts of CD3^+^ (**C**), CD4^+^ (**D**), and CD8^+^ T cells (**E**) in recovered and healthy children. (**F**) The ratio of the CD4^+^/CD8^+^ cell population among different age groups. (**G**) The absolute counts of CD19^+^ B cells in recovered and healthy children. Each dot represents one donor. Data were analyzed using unpaired, two-tailed *t*-tests, *p < 0.05. Error bars represent the mean and SD. RC, recovered children; HC, healthy control; S, spike; N, nucleocapsid.

We also counted and analyzed white blood cells (WBCs), neutrophils, and lymphocytes in the plasma during the infection and recovery phases. The percentages of these cells were within normal limits at the follow-up visit approximately 6–8 months after diagnosis confirmation. No significant lymphocytopenia of the investigated children was observed during the acute infection period (Figure 2A). In addition, we collected PBMCs and applied an 11-color flow cytometry panel (Supplementary Figure S2A), and the PBMCs were divided into seven cell types (Figure 2B). We further classified the two groups of children and projected the data on tSNE maps according to different age groups (Supplementary Figure S2B,C). The distribution of immune cells was similar across the three age groups in recovered or healthy children, but was different between these two populations. To further quantify this difference, we performed absolute quantification of immune cells in the samples. There were no differences in T cells (CD14-CD56-CD3^+^) among PBMCs between healthy children and recovery phase individuals. Further analysis of CD3^+^ T cells showed that there was no significant difference in the magnitude or frequency of CD4^+^ or CD8^+^ T cells or in the CD4^+^/CD8^+^ ratio (Figure 2C–F). Further analysis showed that there was a significant difference in the proportion of B cells (CD3-CD19^+^) between healthy and recovered children in the lower age groups (0–4 and 5–9), with a lower level in infected children, while no significant difference was observed in the 10–14-year-old group (Figure 2G). In brief, except for B cells in 0-to-9-year-old children, no significant lymphopenia was observed in recovered children, and the number of adaptive immune cells in the blood of recovered patients returned to homeostasis 6–8 months after acute infection.

### 3.4. SARS-CoV-2-Specific Memory T Cells Could Be Detected in Recovered Children

**Figure 3:**
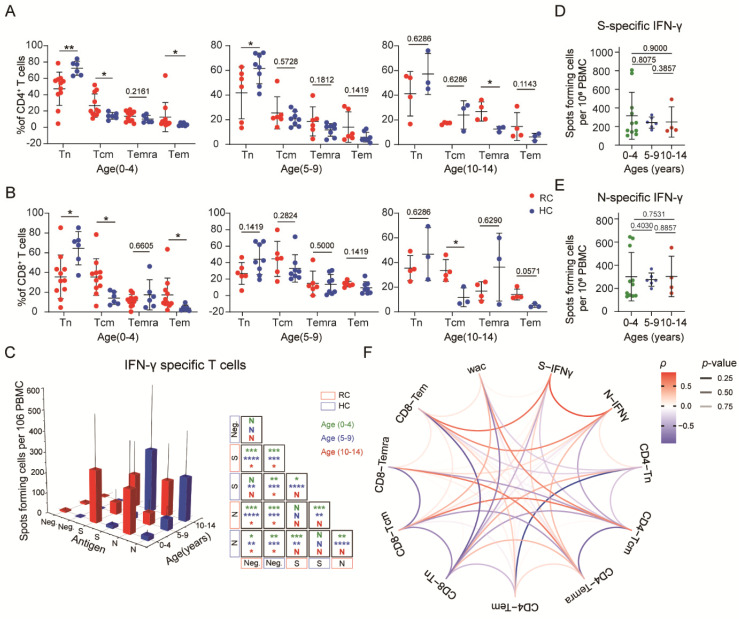
SARS-CoV-2-specific memory T cells were detected in children with mild COVID-19: (**A**) Comparisons of the frequency of helper T cell immune memory subsets in different age groups. (**B**) Comparisons of the frequencies of cytotoxic T cell immune memory subsets in different age groups. (**C**) Spike- and nucleocapsid-specific IFN-γ memory T cell counts in recovered and healthy children. (**D**,**E**) Comparisons of spike- and nucleocapsid-specific IFN-γ memory T cell counts by age category in recovered and healthy children. (**F**) Correlations among the frequencies of different memory T cell subsets, WAC and IFN-γ responses. Red indicates a positive correlation, blue indicates a negative correlation, and transparency represents a change in the *p* value; the greater the *p*-value is, the greater the line transparency. Each dot represents one donor. Data were analyzed using unpaired, two-tailed *t*-tests (ns, *p* > 0.05, * *p* < 0.05, ** *p* < 0.01, and *** *p* < 0.001, and **** *p* < 0.0001). Error bars represent the mean and SD. RC, recovered children; HC, healthy control; WAC, weeks after confirmation; S, spike; N, nucleocapsid.

We next investigated whether immune memory T cells exist in the peripheral blood of recovered children. CD4^+^ T cell and CD8^+^ T cell populations were divided into naive (CD45RA+CD27+CCR7+), central memory (CD45RA−CCR7+ (CM)), effector memory (CD45RA−CCR7− (EM)), and effector memory CD45RA+ (EMRA) (CD45RA+CCR7−) T cells. Both CD4^+^ and CD8^+^ T cell levels showed a significant increase in central memory (Tcm) and effector memory (Tem) cell proportions in recovered children in the 0-to-4-year-old groups. However, in the 5-to-9-year-old groups, no significant differences in memory cell levels were found. Furthermore, the frequencies of Temra cells among CD4^+^ cells and of Tcm cells among CD8^+^ cells were increased in the 10-to-14-year-old group (Figure 3A,B). In addition, no difference in the proportion of naive T and memory T cell subtypes was discovered in children of different ages recovered from mild COVID-19 (Supplementary Figure S2D).

To validate SARS-CoV-2-specific memory T cell immunity in COVID-19 convalescent children, we synthesized S and N protein to stimulate PBMCs. We found that the expression of IFN-γ in T cells from recovered children was significantly increased after stimulation with the S or N protein compared with that in the control cells. In the 0–4-year-old and 5–9-year-old age groups, the response of healthy children was much lower than that of recovered children, yet there was no such difference in the 10–14-year-old age group. We also found a low-intensity response to S or N protein in nonexposed individuals, which reveals cross-reactivity with other common circulating coronaviruses (Figure 3C). No difference was observed in the response to S or N protein. Taken together, these data indicated that memory T cells specific for the S or N protein were generated and sustained in the peripheral blood of COVID-19 convalescent children, with no significant difference in IFN-γ response degree against second exposure among different ages (Figure 3D,E).

To examine interrelations among the immune memory compartments and associations between the memory cell levels and COVID-19 recovery time, we performed comprehensive sets of correlation analyses. We observed a strong positive correlation between S/N-specific IFN-γsecretion and CD8^+^ Tem cells but a negative correlation with CD8^+^ Tn and CD4^+^ Tcm cells. Additionally, the frequencies of CD4^+^ Tn and CD8^+^ Tn cells were decreased over time after diagnosis confirmation, yet stabilization of total CD3^+^ T cells was observed in recovered children, suggestive of depleted naive T cells gradually being replaced by memory T cells. Indeed, we observed a positive correlation between memory T cell frequencies and weeks after diagnosis confirmation, especially for CD4^+^ Tcm and CD8^+^ Tcm cells (Figure 3F).

### 3.5. SARS-CoV-2-Specific Memory B Cells Were Less Responsive in Recovered Children

**Figure 4:**
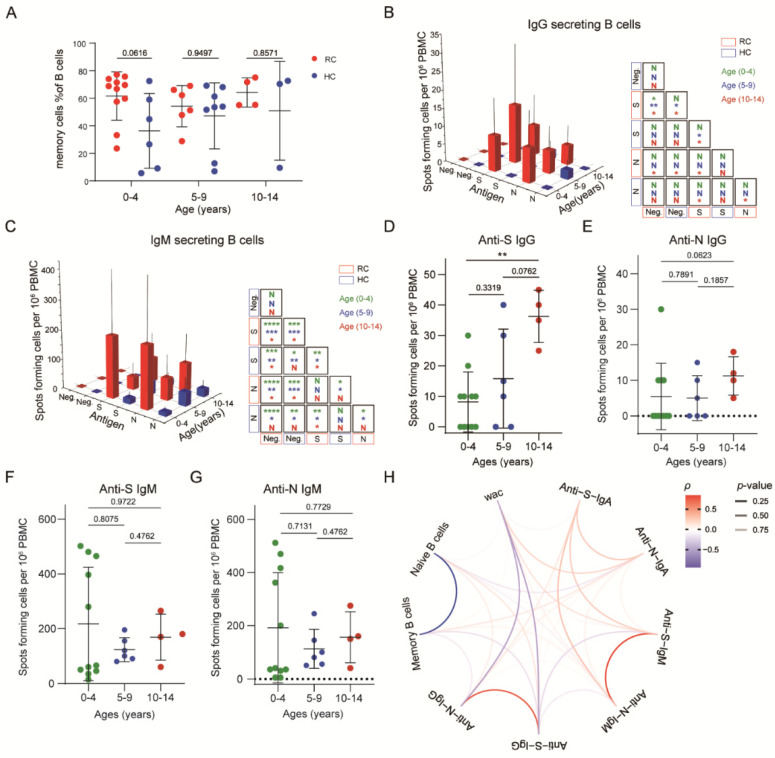
SARS-CoV-2-specific memory B cells show a weak response in children with mild COVID-19: (**A**) Comparisons of the memory B cell frequency of recovered and healthy children in different age groups. (**B**,**C**) Spike- and nucleocapsid-specific IgG^+^, IgM^+^ and memory B cell counts in recovered and healthy children. (**D**–**G**) Comparisons of spike- and nucleocapsid-specific IgG^+^, IgM^+^ memory B cell counts by age category in recovered and healthy children. Each dot represents one donor. (**H**) Comparisons of spike- and nucleocapsid-specific antibody memory B cell numbers by age category in recovered and healthy children. Red indicates a positive correlation, blue indicates a negative correlation, and transparency represents a change in the *p* value; the greater the *p* value is, the greater the line transparency. Data were analyzed using unpaired, two-tailed t-tests (ns, *p* > 0.05, * *p* < 0.05, ** *p* < 0.01, and *** *p* < 0.001, and **** *p* < 0.0001). Error bars represent the mean and SD. RC, recovered children; HC, healthy control; WAC, weeks after confirmation; S, spike; N, nucleocapsid.

Memory B cell subpopulations were also detected in the recovered children. We observed that there were no significant differences in memory B cell (CD19^+^CD27^+^) frequency between recovered and healthy children at any age (Figure 4A).

To identify whether SARS-CoV-2–specific memory B cells exist, we used different fluorescently labeled IgG, IgM, and IgA probes to detect B cells responsive to S or N protein stimulation. Unlike the memory T cell response, IgG^+^-specific memory B cells in SARS-CoV-2–unexposed individuals were rare (S:1/21; N:2/21). Compared with these children, children from the recovered group showed higher specific memory B cell frequencies. The response rates for S protein in the 0-to-4, 5-to-9, and 10-to-14-year-old groups were 54.5%, 66.7%, and 100%, respectively. For the N protein, the response rates were 27.2%, 50%, and 100%, respectively. Among the 0-to-4-year-old group, there was no significant difference in the IgG^+^-specific memory B cell response between healthy and recovered children. The S-specific memory B cells of recovered children were significantly more abundant than those of healthy donors in the 5-to-9-year-old groups. Especially in the 10-to-14-year-old groups, both S- and N-specific memory B cells of recovered children were abundant. Unexpectedly, we found a robust IgM^+^ memory B cell response among children who recovered from COVID-19 in all age groups, while IgA^+^ responses occurred in the 0–4-year-old and 10–14-year-old age group. Meanwhile, there was no significant difference between the IgM^+^ and IgA^+^ memory B cell response to the S or N protein, while IgG^+^ memory B cells responded more strongly to the S protein (Figure 4B,C, Supplementary Figure S3A). Overall, the number of S-specific IgG^+^ memory B cells that could respond to the specific antigen of SARS-CoV-2 increased with age while that did not find in IgM^+^ and IgA^+^ responses. (Figure 4D–G, Supplementary Figure S3B,C). The secondary response to antibodies indicated the presence of SARS-CoV-2-specific memory B cells in recovered children.

We also analyzed the relationships among antibody secretion, the memory B cell level, and the weeks after diagnosis confirmation. Intriguingly, we found that the IgG response was negatively correlated with the weeks after diagnosis confirmation and the memory B cell level, while the IgM response showed a positive correlation. Furthermore, the S-specific IgG response was negatively correlated with the S-specific IgM response (Figure 4H). In summary, the results showed a more robust IgM^+^ memory B cell response than IgG^+^ response, and the number of S-specific IgG^+^ memory B cells increased with age.

## 4. Discussion

The severity of clinical symptoms and prognosis of children infected with SARS-CoV-2 have always been the focus of research on SARS-CoV-2 infection and immunization [16,17]. Insight into whether children can acquire immune memory post-infection and the extent to which they can recover during recovery is crucial to evaluating the value of childhood passive immunization and improving treatment options for children [18,19]. Clinical manifestations showed that although some mild or asymptomatic children present respiratory symptoms accompanied by increased inflammatory markers in the acute infection phase, most of them recover within 6–8 months. Unfortunately, although the recovered patients avoided fatigue, mental stress, and other conditions to prevent secondary infection, and maintained oxygen inhalation and carried out lung function recovery training, it was found that the lesions of some patients were not completely recovered and their lung function failed to reach a healthy level during the lung function test. In contrast with healthy children, we determined that there was still a certain level of antibody in serum from recovered subjects. Moreover, the immune system reattains healthy status, forming immune memory and producing an enhanced memory response to secondary SARS-CoV-2 antigen stimulation.

These results extend studies on immune memory in humans after coinfection with SARS-CoV-2 infection, demonstrating that immune memory is also present in children [20,21]. In addition, our findings suggested that childhood immunity is not the same as adult immunity [22]. In children who recovered, antibody levels increased faster and more markedly over time, while in adults, they declined more slowly [23]. We also found higher IgM cutoff values in systematic serological tests of children, which is consistent with the conclusion of W. Chung et al. [22]. This finding could mean that IgM, as a relatively sensitive antibody, is better able to protect against the virus once it invades, leading to a higher proportion of mild disease in children.

In addition to the differences in antibody protection, there were also significant differences in cellular immunity. In combination with the work of Nimesh Gupta et al., we found that the response of IFN-γ-secreting CD4^+^ and CD8^+^ T cells to viral structural proteins in recovered children was significantly less than that in adults [24,25,26]. Furthermore, Shane Crotty et al. identified that adult memory B cells mainly secrete IgG-subtype antibodies in response to SARS-CoV-2 [27,28]. However, our study showed a higher proportion of IgM than IgG to SARS-CoV-2 S or N protein stimulation in children; that is, in contrast to memory B cells producing IgG, the child immune response tended to consist of IgM^+^ memory B cells differentiating and proliferating more rapidly in response to SARS-CoV-2 invasion, suggesting that characteristics of memory B cells in children are different from those in adults. Antonio Di Sabatino et al. reported that the loss of IgM^+^ memory B cells in adults is usually indicative of a poor prognosis in SARS-CoV-2 patients, which also provides evidence to explain the milder clinical outcome of SARS-CoV-2 infection in children than in adults [29,30,31].

Compared with asymptomatic or mild patients, severe children patients exhibited more obvious symptoms of peripheral blood lymphocytopenia and increased inflammatory factor levels [32,33]. Therefore, the study of children has mainly concentrated on severe disease and its complications, with scant reports specifically focused on asymptomatic or mildly symptomatic patients, especially for the recovery of bodily function and immune memory. Limited by the investigation population, our study recruited only asymptomatic or mildly infected children and found no history of allergic diseases such as asthma or dermatitis. Children with these diseases often carry auto-antibodies, which may worsen an illness after infection with SARS-CoV-2, or even evolve into multiple systems inflammatory syndrome in children (MIS-C) [33,34]. Similarly, IgG antibody level was positively correlated with the severity of the disease, which was also confirmed by our results. In mild patients who recovered from the disease, IgG disappeared for a period of time after recovery, and memory B cells replaced the antibodies in plasma to protect the body [35,36]. In general, this study showed that lung function in infants takes more time to recover, and we also proved that the immune system in children returned to normal 6–8 months after SARS-CoV-2 infection and established SARS-CoV-2-specific memory T cell and B cell responses.

From our results, it was confirmed that children diagnosed with mild symptoms have sustained antibody responses after infection. Notably, they also produced T cell and B cell responses to the viral spike and nucleocapsid proteins, even though the response was less intense than that in adults [37]. In conclusion, our results suggest that children with mild symptoms can form systemic immune memory and prevent severe infection to some extent, thus contributing to the establishment of herd immunity.

## Figures and Tables

**Table 1:** Clinical features of children recovered from mild COVID-19.

Clinical Indicators	0–4 Years Old (%)	5–9 Years Old (%)	10–14 Years Old (%)
**Clinical type**			
Asymptomatic	1 (6%)	0 (0%)	1 (17%)
Acute Upper Respiratory Infection (AURI)	5 (31%)	4 (44%)	2 (33%)
Mild pneumonia	10 (63%)	5 (56%)	3 (50%)
**Symptoms**			
Fever	8 (50%)	4 (44%)	3 (50%)
Cough	12 (75%)	4 (44%)	3 (50%)
Expectoration	4 (25%)	1 (13%)	0 (0%)
Congestion	2 (13%)	0 (0%)	0 (0%)
Excessive fatigue	0 (0%)	0 (0%)	1 (17%)
**CT findings (Initial diagnosis)**			
Normal	6 (38%)	4 (44%)	3 (50%)
Ground-glass Opacity (GGO)	7 (44%)	3 (33%)	2 (33%)
Patchy Shadow	3 (19%)	2 (22%)	1 (17%)
**Pneumonia**			
NA	6 (38%)	4 (44%)	3 (50%)
Unilateral (Left)	2 (13%)	0 (0%)	0 (0%)
Unilateral (Right)	3 (19%)	3 (33%)	1 (17%)
Bilateral (Both)	5 (31%)	2 (22%)	2 (33%)
**CT findings (Follow-up visit)**			
Not Recovered	8 (50%)	3 (33%)	0 (0%)

No signs of meaningful complications were observed. The proportion shown in the table is that of the characteristic in the total population of the corresponding age group.

## Data Availability

Further information and requests for resources and reagents should be directed to J.L.

## Supplementary Materials

The following supporting information can be downloaded at: https://www.mdpi.com/article/10.3390/v14010085/s1, Figure S1: SARS-CoV-2-specific IgM response in children with mild COVID-19 at 6–8 months after infection, Figure S2: Phenotype analysis of immune cells, Figure S3: SARS-CoV-2-specific IgA^+^ memory B cells with mild COVID-19, Table S1: Age and sex statistics of experimental group samples, Table S2: Serological markers of serum samples from follow-up children, Table S3: Pulmonary function test values of recovered children at revisit.
