# Supplementary material for: Evaluation of Clinical and Immune Responses in Recovered Children with Mild COVID-19
Source: Viruses. 2022 Jan 4;14(1):85. doi: 10.3390/v14010085 (PMC8779549; doi:10.3390/v14010085)
Supplement: Supplementary file 1 [file viruses-14-00085-s001.zip › viruses-1513918-supplementary.pdf]

Supplementary Figures

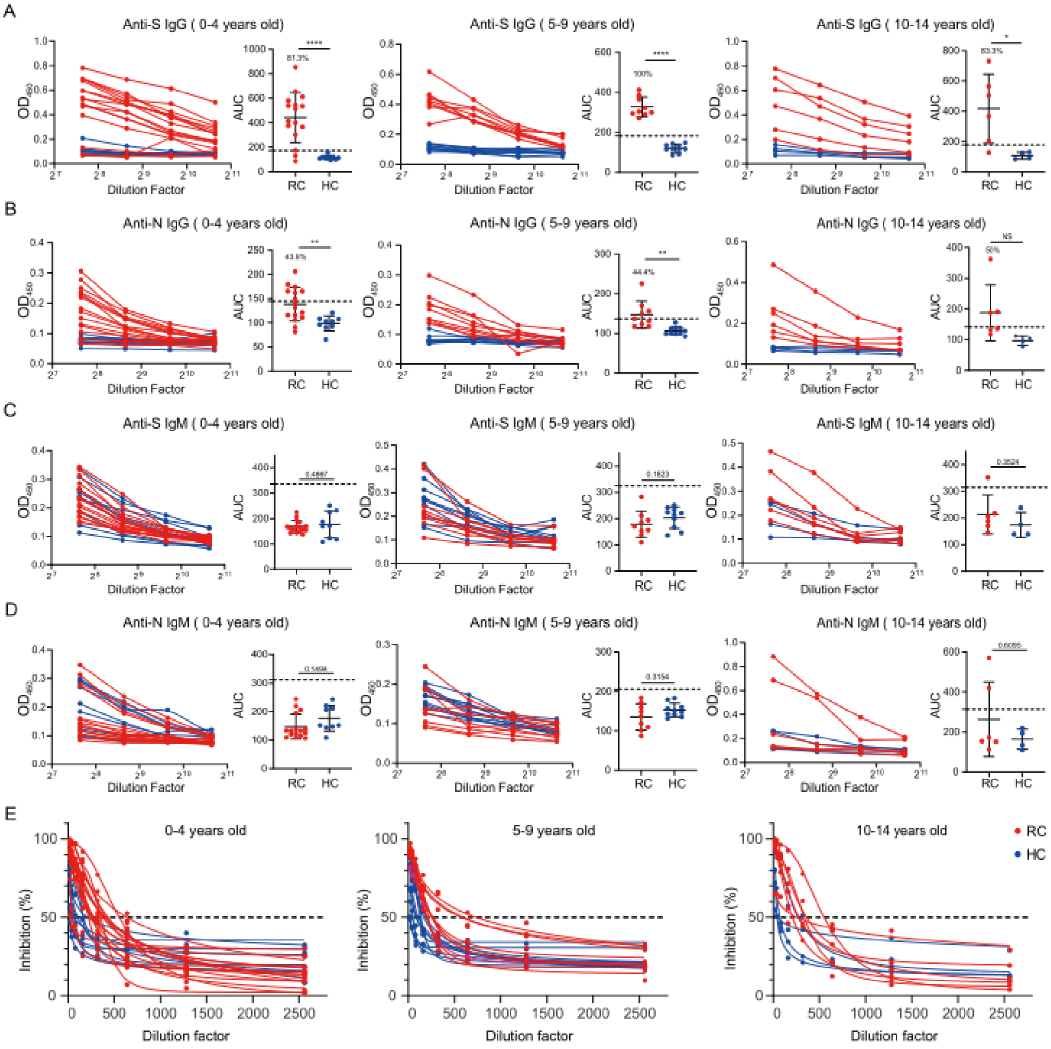

Figure S1. SARS-CoV-2-specific IgM response in children with mild COVID-19 at 6-8 months after infection. (A-D) ELISA dilution curves and area under the curve (AUC) for anti-S/N IgG and IgM in different age groups. HC, n=22; CP, n=31. The dashed line indicates the mean + 3 SD of the HC AUC values. The significant differences between the groups are marked by bars. (E) Serum neutralizing antibody dilution curves in different age groups. Data were analyzed using unpaired, two-tailed t-tests, ns, $p > 0.05$, *$p < 0.05$, **$p < 0.01$, and ****$p < 0.0001$. Error bars represent the mean and SD. RC, recovered children; HC, healthy control; S, spike; N, nucleocapsid.

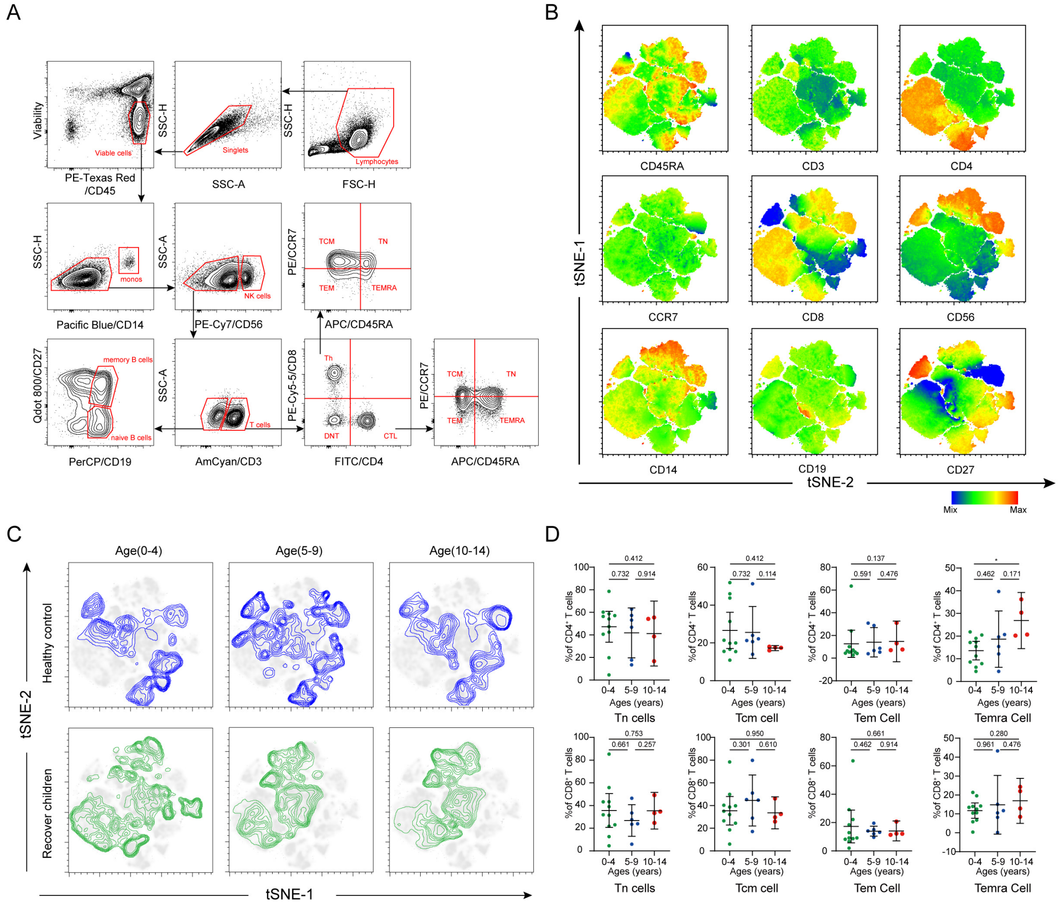

Figure S2. Phenotype analysis of immune cells. (A) Flow cytometry plots shows the gating strategy. (B) Projection of the expression levels of different markers on tSNE plots. (C) Projection of cell clusters in different age groups of healthy and recovered children. (D) Comparison of frequency of naive T cells and memory T cell subsets of different age categories in recovered and healthy children. Data were analyzed using unpaired, two-tailed t-tests (ns, $p > 0.05$, $^{*}p < 0.05$)

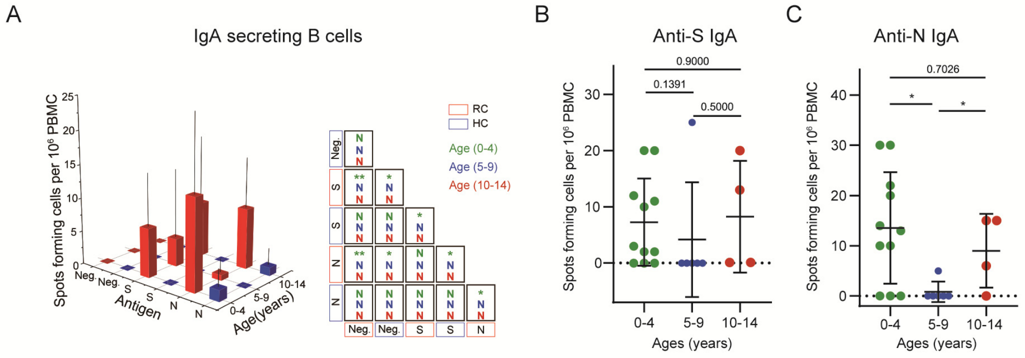

Figure S3. SARS-CoV-2-specific IgA+ memory B cells with mild COVID-19. (A) Spike- and nucleocapsid-specific IgA+ memory B cell counts in recovered and healthy children. (B, C) Comparison of spike- and nucleocapsid-specific IgA+ memory B cells by age category in recovered and healthy children. Each dot represents one donor. Data were analyzed using unpaired, two-tailed t-tests (ns, $p > 0.05$, *$p < 0.05$)

Supplementary Tables

Table S1. Age and sex statistics of experimental group samples

Groups	Healthy control		Recover children		
	Ages (mean ±STDEV)	Male (%)	Ages (mean ±STDEV)	Male (%)	Recovered weeks
0-4 years old	1.56 ± 1.13	5 (56%)	1.79 ± 1.55	8 (50%)	31.25 ± 1.68
5-9 years old	6.50 ± 1.12	5 (50%)	7.80 ± 0.94	8 (89%)	30.67 ± 0.67
10-14 years old	12.00 ± 0.71	4 (100%)	11.87 ± 0.72	4 (67%)	29.67 ± 2.36
Average	5.52 ± 3.87	14 (61%)	5.64 ± 4.56	20 (65%)	30.77 ± 1.74

Table S2. Serological markers of serum samples from follow-up children (n=31)

Serological markers	0-4 years old Mean (range)	5-9 years old Mean (range)	10-14 years old Mean (range)
Alanine Aminotransferase (ALT, U/L)	25.88 (8-66)	12.50 (9-16)	41.00 (6-101)
Aspartate Aminotransferase (AST, U/L)	41.75 (21-75)	24.00 (20-35)	33.67 (14-50)
Lactate Dehydrogenase (LDH, U/L)	297.38 (197-656)	240.00 (177-388)	270.17 (162-363)
Creatinine (μmol/L)	23.11 (17.3-30.4)	36.69 (29-45.2)	42.00 (33.1-46.1)
Creatine kinase (CK, U/L)	138.69 (40-326)	99.50 (79-148)	101.50 (28-138)
Creatine kinase-MB (CK-MB, U/L)	40.69 (20-153)	24.75 (14-47)	30.83 (10-62)
D dimer (ng/mL)	0.39 (0-2.05)	0.99 (0-6.97)	12.61 (0-31.8)
C-Reactive Protein (CRP, mg/L)	5.15 (0-30.3)	0.86 (0-1.27)	12.86 (0-24.8)
Procalcitonin (PCT, μg/L)	0.07 (0.04-0.15)	1.37 (0-9.25)	0.16 (0.05-0.43)

Table S3. Pulmonary function test values of recovered children at revisit

Pulmonary function test values	0-4 years old (Mean)	5-9 years old (Mean)	10-14 years old (Mean)
VCMAX	24.48	92.43	81.52
FVC	38.75	95.10	81.90
FEV1	19.50	101.82	89.50
FEV1/FVC	20.03	106.26	108.88
PEF	17.48	94.71	85.00
FEF25	36.49	100.04	93.46
FEF50	37.75	98.12	95.38
FEF75	96.52	92.04	100.38
MMEF75/25	86.77	97.76	96.58
Conclusion			
NA	10 (63%)	9 (100%)	4 (66%)
Mildly restrictive lesion	0 (0%)	0 (0%)	2 (33%)
Mild to moderate obstructive disease	2 (13%)	0 (0%)	0 (0%)
Moderate obstructive disease	4 (25%)	0 (0%)	0 (0%)
